# Detecting the Feeble Electromagnetic Emissions from Cancer Biomarkers

**DOI:** 10.1021/acscentsci.5c00556

**Published:** 2025-04-09

**Authors:** Marcos Dantus

**Affiliations:** † Department of Chemistry, Michigan State University, East Lansing, Michigan 48824, United States; ‡ Department of Physics and Astronomy, Michigan State University, East Lansing, Michigan 48824, United States; § Department of Electric and Computer Engineering, Michigan State University, East Lansing, Michigan 48824, United States

In 1966, Gene Roddenberry’s *Star Trek* introduced a futuristic device called the tricorder, capable of noninvasively diagnosing diseases. Early cancer detection, which significantly impacts prognosis, could certainly benefit from a tricorder, and has spurred the development of multiple non- or minimally invasive diagnostic methods.

In 2024, these cancers accounted for 20%, 2.8%, 14.5%, and 9.6% of cancer deaths in the US, respectively. The percentages for breast cancer are for the female population, while those for prostate cancer are for the male population.^1^

The use of diagnostic tools dates back to 400 BCE, when Hippocrates claimed disease could be detected through smell. Over the centuries, devices like the stethoscope and otoscope have played key roles in diagnosis for more than two centuries. In the 21st century, laboratory tests, along with imaging technologies like MRI (Magnetic Resonance Imaging), CT (Computed Tomography) scans, and PET (Positron Emission Tomography) scans, have become essential in disease diagnosis and are considered the gold standard. Advances in disease biomarker detection, such as selective biochemical binding seen in pregnancy tests and COVID-19 diagnostics, have further enhanced diagnostic capabilities. However, similar methods for early cancer detection remain underdeveloped. Cell-free DNA fragmentation in cancer patients shows promise as a potential target for cancer screening and early detection, although it remains a time-consuming approach.^2^

Zigman’s article focuses on the time-dependent response of blood plasma to ultrashort laser pulses. The underlying principle can be understood through an acoustic analogy: molecules vibrate at different frequencies, which can be detected.

Despite individual variability, Zigman’s study successfully demonstrates the ability to distinguish patients with breast, bladder, lung, and prostate cancer, which collectively account for a significant proportion of cancer-related deaths.

Zigman’s method, electric-field molecular fingerprinting (EMF) shown in Figure 1, differs from traditional IR absorption, in that the mid-IR broadband source (910–1530 cm^–1^) is delivered as a femtosecond pulse that passes through the sample, and a second femtosecond pulse is used to convert the signal through electrooptic sampling into a time-dependent polarization.^3^ The signal is analogous to the simultaneous striking of many bells, each ringing at the same time but producing distinct sounds. Zigman’s method shares similarities with coherent Raman spectroscopy,^4,5^ differing primarily in how the temporal data is transformed into the frequency domain. The EMF method directly analyzes the time-domain signal, similar to Fourier transform IR spectroscopy, whereas coherent Raman spectroscopy often collects the signal in the frequency domain.

**Figure 1:**
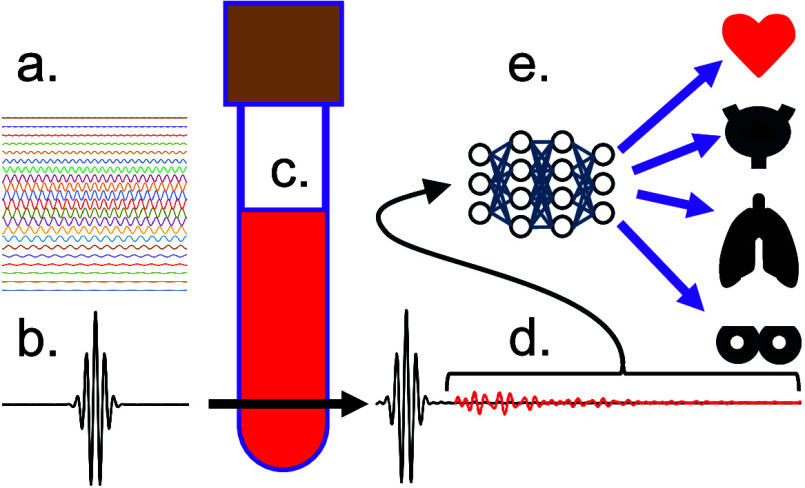
Cancer detection using electric-field molecular fingerprinting of blood plasma. A broadband femtosecond pulse (a) is transmitted through the plasma (c). The transmitted beam (b) exhibits a residual signal (d) that depends on the vibrational absorption by the plasma components. This signal is then analyzed using machine learning (e) to assess the likelihood that the plasma sample is from a healthy individual or one with bladder, lung, prostate (not shown), or breast cancer.

The collected signal is processed by a machine learning model, trained on a set (80% of individuals) and validated against an independent test set (20% of the individuals). Performance is evaluated based on discrimination between true positives and false positives for each of the cancer types, with a scale where 0.5 represents random performance and 1.0 indicates perfect distinction between the two classes. The training set results were 0.88 for lung cancer, 0.68 for prostate cancer, 0.69 for breast cancer, and 0.68 for bladder cancer. The independent set results were 0.81 for lung cancer, 0.71 for prostate cancer, 0.57 for breast cancer, and 0.58 for bladder cancer. The ability to discriminate among lung, prostate, and bladder cancers is found to be 0.48 and 0.53 for female and male cohorts, respectively. These results are significant, given that with three options, random guessing would yield a value of 0.33.

Notably, the method demonstrates robust results, particularly for advanced-stage lung cancer, with values of 0.6, 0.8, 0.87, and 0.92 for stages I through IV, respectively. These findings suggest a dose–response relationship that further supports the ability of EMF to detect lung cancer biomarkers. Further improvements in sensitivity should enhance the method’s ability to identify lung cancer and other cancers in their earliest stages.

EMF detection has been found to be comparable to infrared (IR) absorption, which has been studied for much longer.^6^ Detecting vibrational biomarkers often involves infrared spectroscopy, as well as advanced laser methods like Raman and coherent Raman spectroscopy.^7,8^ Among these, the sensitivity of the technique and the sophistication of data analysis play a significant role in results. Variations in individuals (e.g., age, gender, health, nutrition, medications) can also impact results, as can the extraction and preparation of blood samples. Another exciting application of these methods is the histopathologic identification of cancer and determining tumor resection margins, an area where vibrational spectroscopy is making significant progress.^9,10^

The results of this study justify further efforts to improve the laser system, data acquisition, and analysis methods to achieve an acceptable level of false positives and negatives, and to support its use as a minimally invasive screening approach. As the technology develops, testing on larger populations will be necessary to further validate the approach.

However, for this to become a reality, the technology must evolve to offer fast, accurate, and cost-effective analysis.

## References

[ref1] Kepesidis K. V.; et al. Electric-field molecular fingerprinting to probe cancer. ACS Central Science 2025, 10.1021/acscentsci.4c02164. PMC12022918 40290141

[ref2] Cristiano S.; Leal A.; Phallen J.; Fiksel J.; Adleff V.; Bruhm D. C.; Jensen S. Ø.; Medina J. E.; Hruban C.; White J. R.; Palsgrove D. N.; Niknafs N.; Anagnostou V.; Forde P.; Naidoo J.; Marrone K.; Brahmer J.; Woodward B. D.; Husain H.; van Rooijen K. L.; Ørntoft M. B. W.; Madsen A. H.; van de Velde C. J. H.; Verheij M.; Velculescu V. E.; et al. Genome-wide cell-free DNA fragmentation in patients with cancer. Nature 2019, 570, 385–389. 10.1038/s41586-019-1272-6. 31142840 PMC6774252

[ref3] Voronina L.; Fleischmann F.; Simunovic J.; Ludwig C.; Novokmet M.; Zigman M. Probing blood plasma protein glycosylation with infrared spectroscopy. Anal. Chem. 2024, 96 (7), 2830–2839. 10.1021/acs.analchem.3c03589. 38324652 PMC10882574

[ref4] Cheng J. X.; Xie X. S. Vibrational spectroscopic imaging of living systems: An emerging platform for biology and medicine. Science 2015, 350, 6264 10.1126/science.aaa8870. 26612955

[ref5] Camp C. H.; Cicerone M. T. Chemically sensitive bioimaging with coherent Raman scattering. Nat. Photonics 2015, 9, 295–305. 10.1038/nphoton.2015.60.

[ref6] Gajjar K.; Trevisan J.; Owens G.; Keating P. J.; Wood N. J.; Stringfellow H. F.; Martin-Hirsch P. L.; Martin F. L. Fourier-transform infrared spectroscopy coupled with a classification machine for the analysis of blood plasma or serum: a novel diagnostic approach for ovarian cancer. Analyst 2013, 138, 3917–3926. 10.1039/c3an36654e. 23325355

[ref7] Bonifacio A.; Cervo S.; Sergo V. Label-free surface-enhanced Raman spectroscopy of biofluids: fundamental aspects and diagnostic applications. Anal. And Bioanal. Chem. 2015, 407, 8265–8277. 10.1007/s00216-015-8697-z. 25935674

[ref8] Kong K.; Kendall C.; Stone N.; Notingher I. Raman spectroscopy for medical diagnostics — From in-vitro biofluid assays to in-vivo cancer detection. Ad. Drug Delivery Rev. 2015, 89, 121–134. 10.1016/j.addr.2015.03.009. 25809988

[ref9] Tu H. H.; Liu Y.; Turchinovich D.; Marjanovic M.; Lyngso J. K.; Lægsgaard J.; Chaney E. J.; Zhao Y. B.; You S. X.; Wilson W. L.; Xu B. W.; Dantus M.; Boppart S. A. Stain-free histopathology by programmable supercontinuum pulses. Nat. Photonics 2016, 10, 534 10.1038/nphoton.2016.94. 27668009 PMC5031149

[ref10] Bhargava R. Digital Histopathology by Infrared Spectroscopic Imaging. Annu. Rev. Anal. Chem. 2023, 16, 205–230. 10.1146/annurev-anchem-101422-090956. PMC10408309 37068745

